# Cardiometabolic risks and atherosclerotic disease in ApoE knockout mice: Effect of spinal cord injury and Salsalate anti-inflammatory pharmacotherapy

**DOI:** 10.1371/journal.pone.0246601

**Published:** 2021-02-24

**Authors:** Gregory E. Bigford, Angela Szeto, John Kimball, Edward E. Herderick, Armando J. Mendez, Mark S. Nash

**Affiliations:** 1 Department of Neurological Surgery, University of Miami Miller School of Medicine, Miami, Florida, United States of America; 2 Department of Medicine, University of Miami Miller School of Medicine, Miami, Florida, United States of America; 3 EEH, LLC, Pickerington, Ohio, United States of America; 4 Department of Physical Medicine and Rehabilitation, University of Miami Miller School of Medicine, Miami, Florida, United States of America; 5 Department of Physical Therapy, University of Miami, Coral Gables, Florida, United States of America; Max Delbruck Centrum fur Molekulare Medizin Berlin Buch, GERMANY

## Abstract

**Objective:**

To test in mice with a double mutation of the ApoE gene (*ApoE*^*-/-*^) whether spinal cord injury (SCI) hastens the native trajectory of, and established component risks for, atherosclerotic disease (AD), and whether Salsalate anti-inflammatory pharmacotherapy attenuates the impact of SCI.

**Methods:**

*ApoE*^*-/-*^ mice were anesthetized and underwent a T9 laminectomy. Exposed spinal cords were given a contusion injury (70 k-dynes). Sham animals underwent all surgical procedures, excluding injury. Injured animals were randomized to 2 groups: SCI or SCI_+Salsalate_ [120 mg/Kg/day i.p.]. Mice were serially sacrificed at 20-, 24-, and 28-weeks post-SCI, and body mass was recorded. At sacrifice, heart and aorta were harvested intact, fixed in 10% buffered formalin, cleaned and cut longitudinally for *en face* preparation. The aortic tree was stained with oil-red-O (ORO). AD lesion histomorphometry was calculated from the proportional area of ORO. Plasma total cholesterol, triglycerides and proatherogenic inflammatory cytokines (PAIC’s) were analyzed.

**Results:**

AD lesion in the aortic arch progressively increased in *ApoE*^*-/-*^, significant at 24- and 28-weeks. AD in SCI is significantly greater at 24- and 28-weeks compared to time-controlled *ApoE*^*-/-*^. Salsalate treatment attenuates the SCI-induced increase at these time points. Body mass in all SCI groups are significantly reduced compared to time-controlled *ApoE*^*-/-*^. Cholesterol and triglycerides are significantly higher with SCI by 24- and 28-weeks, compared to *ApoE*^*-/-*^, and Salsalate reduces the SCI-induced effect on cholesterol. PAIC’s interleukin-1β (IL-1β), interleukin-6 (IL-6), tumor necrosis factor α (TNFα), monocyte chemoattractant protein-1 (MCP-1), and chemokine (C-C motif) ligand 5 (CCL-5) are significantly greater with SCI compared to *ApoE*^*-/-*^ at varying timepoints. Salsalate confers a marginal reducing effect on PAIC’s by 28-weeks compared to SCI. Regression models determine that each PAIC is a significant and positive predictor of lesion. (p’s <0.05).

**Conclusions:**

SCI accelerates aortic AD and associated risk factors, and anti-inflammatory treatment may attenuate the impact of SCI on AD outcomes. PAIC’s IL-1β, IL-6, TNFα, MCP-1, and CCL-5 may be effective predictors of AD.

## Introduction

Chronic spinal cord injury (SCI) results in a greater prevalence of risk factors for cardiovascular disease (CVD) and atherosclerotic disease (AD) when compared to the able-bodied population. These previously reported risks include central obesity [1–5], fasting dyslipidemia [6–8], hypertension (in persons with paraplegia) [5,7,8], and insulin resistance derived from the homeostatic model 2 (HOMA2) method or quantitative insulin sensitivity check index (QUICKI) [7,9,10]. Other established AD risk factors reported after SCI include physical deconditioning [11–14], postprandial lipemia [15,16], and inflammatory vascular stress [16–18]. These risks have an alarming tendency to cluster in persons with SCI [7,19], which escalates the global AD vulnerability of the population [7,20].

These widely reported AD risk factors after SCI raise a fundamental question whether–or to what extent–SCI alters the trajectory of atherogenesis and vascular system pathology. AD has long been viewed as a ‘lipid storage disease’ [21] and in this regard, It would be obvious to suggest that ‘dyslipidemia’ after SCI is uniquely responsible for AD risk in the population [10,22]. However, it is now better understood that narrowing of the arteries does not necessarily predict ‘hard disease’ (i.e. myocardial infarction), and that AD still evolves in persons having unremarkable lipid profiles, where nearly half of the cases involving symptomatic AD are unexplained by dysplipidemia [23–26]. More contemporary approaches to AD diagnosis and clinical management have focused on AD as an inflammatory disorder defined by the presence and actions of pro-atherogenic inflammatory cytokines (PAIC) [27,28]. whose integration into traditional lipid prediction models improves the forecasting of future AD [29], and whose targeted treatment decreases hard cardiac events [24,25]. While there are several reports of significantly elevated levels of PAIC after SCI [17,18], their role in stimulating atherogenesis remains unresolved. A clearer explication of risks that underlie AD progression after SCI is required if we are to understand who is at risk and how to undertake effective intervention.

A critical regulatory mechanism of PAIC is activation of IκB kinase complex β (IκKβ)/nuclear factor kappa B (NF-κB) signaling pathways, which mediates the synthesis of several key cytokines implicated in AD and SCI, including TNFα, IL-6, and IL-1β [30]. Substantial evidence supports pathological activation of NF-κB-mediated pathways in cardiometabolic diseases (CMD) including CVD and diabetes [31]. Importantly, the NF-κB axis can be inhibited by the use of non-acetylated salicylates without eliciting adverse effects [32]. The salicylate drug Salsalate has been shown to significantly reduce several inflammatory markers including IL-6 and TNFα [32–35], and ameliorate mechanisms involved in atherosclerosis *in vitro* [36]. and randomized placebo-controlled studies demonstrate that Salsalate was associated with a decreased pro-atherogenic lipid profile [37,38]. The extent of its effects on lipid profile and inflammation in SCI have not been evaluated.

The primary objective of this study is to quantify disease-specific changes–often referred to as ‘hard’ disease–in the aortas of mice that have undergone SCI compared to sham-treated mice. We evaluated lipid profiles and pro-atherogenic inflammatory cytokines as secondary, yet still important objectives, and test the biological effects of Salsalate pharmacotherapy on lipid, inflammatory and AD phenotype observed with SCI.

## Materials and methods

### Grouping and treatments

Four groups of mice began testing at 16 weeks of age. 3 groups: *ApoE*^*-/-*^_*SCI*_, *ApoE*^*-/-*^_*SCI/sal*_, *ApoE*^*-/-*^_SCI/veh_*, underwent contusion injury and 1 group: *ApoE*^*-/-*^_sham_*, sham procedure. A subset of animals from each group was sacrificed in week 20 when visible aortic lesions typically emerge in *ApoE*^*-/-*^ mice, and this schema was repeated in weeks 24, when lesions are more pronounced and 28, when lesions are abundant [39–41]. In addition, a cohort of *ApoE*^*-/-*^ mice were uninjured and untreated and sacrificed at the time points outlined above as a reference of the natural trajectory of AD lesion. Salsalate (Sigma; 120 mg/Kg/day) was dissolved in Dimethyl sulfoxide (DMSO) according to the manufacturer’s instruction and administered via i.p. injection. Pharmacotherapy was given once daily for 1-month following SCI, consistent with recent evidence indicating efficacy in mice [42,43]. (*These treatment and injury control groups showed no behavioral, *biological and/or statistical difference from untreated and uninjured groups*, *and therefore were removed from analysis)*.

### Experimental animals

All animal protocols were approved by the University of Miami Institutional Animal Care and Use Committee and are in accordance with National Research Council guidelines for the care and use of laboratory animals. Animals were group (socially) housed in a temperature- and humidity- controlled rodent vivarium, maintained on a reverse 12-hour light/dark cycle, and given food and water ad libitum. No additional environmental enrichment was provided due to its reported effects on the nervous system, including neurogenesis and levels of inflammation, which may affect experimental outcomes. Animals were acclimated for seven days prior to study experiments, which included being handled daily to get used to human contact and minimize distress. Following surgical procedures, animals were administered buprenorphine (0.1 mg/kg) BID for 3-days and gentamicin (0.5 mg/100 g) daily for 7-days post-surgery and *pro re nata* (PRN) thereafter. Animals were examined twice daily for post-stress health status by observation of activity level, respiratory rate, and general physical condition. Body weight was monitored every other day as an indicator of post-operative health. Excessive weight loss (>20%) and decreased grooming behavior was considered as criteria for early exclusion from the study and/or euthanasia. Physical conditions such as moribund state, dehydration, and anorexia were also considered as criteria for early termination and removal from randomized study group. Euthanasia was carried out by carbon dioxide inhalation according to the recommendations for euthanasia detailed in the 2007 Report of the American Veterinary Medical Association’s (AVMA) Panel on Euthanasia. Euthanasia was performed in a manner to avoid animal distress. The chamber was not pre-filled with gas before placing the animals inside, and the rate of CO_2_ flow into the chamber was slowly increased. Animals were in a deep state of sleep after one minute, then the CO2 flow rate was increased for another four minutes. After CO_2_ exposure, confirmation of termination was accomplished by cervical dislocation.

### Traumatic SCI

Surgeries were performed at the Animal and Surgical Core Facility of the Miami Project to Cure Paralysis As we previously described [42,44,45], contusion injury was induced with the Infinite Horizon Impactor device adapted to the mouse. The infinite Horizon impactor device has been established in producing precise, graded contusion, with reproducible lesion volume and functional outcomes assessed using Basso, Beattie, Bresnahan (BBB) and Basso Mouse Scale (BMS) [46] open-field locomotor rating scales [47]. In brief, adult female ApoE^-/-^ mice (C57BL/6 background [B6.129P2-ApoEtm1UNC/J], 15 weeks old, weight; 22–24g; Jackson Laboratories) were anesthetized with an intraperitoneal injection of ketamine (80±100 mg/kg) and xylazine (10 mg/kg). Complete anesthetization was determined by the lack of a stereotypical retraction of the hind-paw in response to a nociceptive stimulus. Mice were then subjected to a laminectomy at vertebrae T9 and the exposed spinal cord was injured at a predetermined impact force of 70 kdynes (severe injury). Sham-operated animals underwent all surgical procedures, including laminectomy, but their spinal cords were not injured. After surgery, animals were housed separately and treated with subcutaneous lactated Ringer’s solution to prevent dehydration. Manual bladder expression was performed twice daily. As described above, prophylactic antibiotic gentamicin was administered daily for 7 days to prevent urinary tract infections. *We note, this study was limited to female mice as their neurogenic bladder following experiment SCI is more safely ‘expressed’ than in males, and studies support that female mice develop greater disease burden than in males [48]*.

### Body mass

Body mass discriminated to 0.1g was measured on a calibrated analytic balance (Data Weighing Systems) at 16-weeks of age (Baseline: before SCI survival surgery); 20-, 24-, and 28-weeks of age (post-surgery: prior to necropsy).

### Tissue collection

#### Plasma

On the day of sacrifice, mice were fasted for 7 hours, deeply anesthetized with an IP injection of a ketamine/ xylazine cocktail and exsanguinated by cardiac puncture using a heparinized needle. Samples were transferred to an EDTA coated tube and centrifuged (5000 x g, 15min). Plasma was collected, aliquoted and stored at -80°C until assay. Fasting plasma total cholesterol and triglyceride levels were assayed by enzymatic methods (Roche Diagnostics, Indianapolis, IN). PAIC’s were simultaneously analyzed using a multiplex assay consisting of a panel of 23 cytokine/chemokine biomarkers involved in the inflammatory/immune response (MILLIPLEX MAP Mouse Cytokine/Chemokine Magnetic Bead Panel; EMD Millipore, Danvers, MA).

#### Heart and aorta

On the day of sacrifice, and following exsanguination, the heart and aorta were removed intact, stripped of adventitia, and cut longitudinally to expose the area of definable lesion covering the luminal “*en face*” surface, a widely used and accepted method for quantification of atherosclerotic lesions in mice [49,50]. Tissues were stored in 10% buffered formalin for later staining and quantification of AD.

*Quantitation and Statistical Analysis*. *Quantitation of Atherosclerosis*. All histomorphometric procedures were performed blinded to treatment and grouping. The method for preparation of mice aortas and quantification of disease were performed as previously described [51]. Formalin-fixed aortas were stained with oil-red-O, and digitally photographed. A reference aorta template, created from the average size and shape of all the aortas in the sample, was overlaid onto each aorta image. Percent lesion area was calculated from the proportional area of pixels stained with oil-red-O for a given aortic section.

### Analysis

#### Group comparisons

Between group differences were analyzed using main effects and interactions assessed by a 2-factor (Group [*ApoE*^*-/-*^ vs. *ApoE*^*-/-*^_*SCI*_ vs. *ApoE*^*-/-*^_*SCI/sal*_] by Time [week 16 vs. 20 vs. 24 vs. 28]) two-way analysis of variance (ANOVA), followed by Tukey post hoc for multiple comparison and reflect absolute change between all groups (GraphPad, Prism v8.4.2, R Studio v1.2.1335). Data are expressed as mean ± *standard error of the mean*. An *a priori* significance level of p ≤ 0.05 was accepted as different between groups. n = 8–12 for each group, dependent on quality of sample analysis and/or survival, where last observation carry-forward (LOCF) technique was used.

#### Linear regression

Regression analysis was performed to evaluate whether specific inflammatory analytes are significant predictors of heart disease, quantified by aortic lesion area (GraphPad, Prism v8.4.2, R Studio v1.2.1335). For simple linear regression: IL-1β, IL-6, TNFα, MCP-1, and CCL-5 were selected as predictor variables, and aortic plaque (area) as the response variable. Predictor selection was determined on the basis of between group differences observed following multiplex assay analysis and previously report association with AD (both described above). Multiple linear regression was performed using the above predictor variables to determine whether an integrated model could predict plaque lesion area. Variable distribution was assessed for normality and where necessary was logarithmically transformed. Normality, complete descriptive statistics, ANOVA main effect and interactions, and regression model statistics and diagnostics are summarized in supporting figures and tables.

## Results

### Atherosclerotic disease

**Fig 1:**
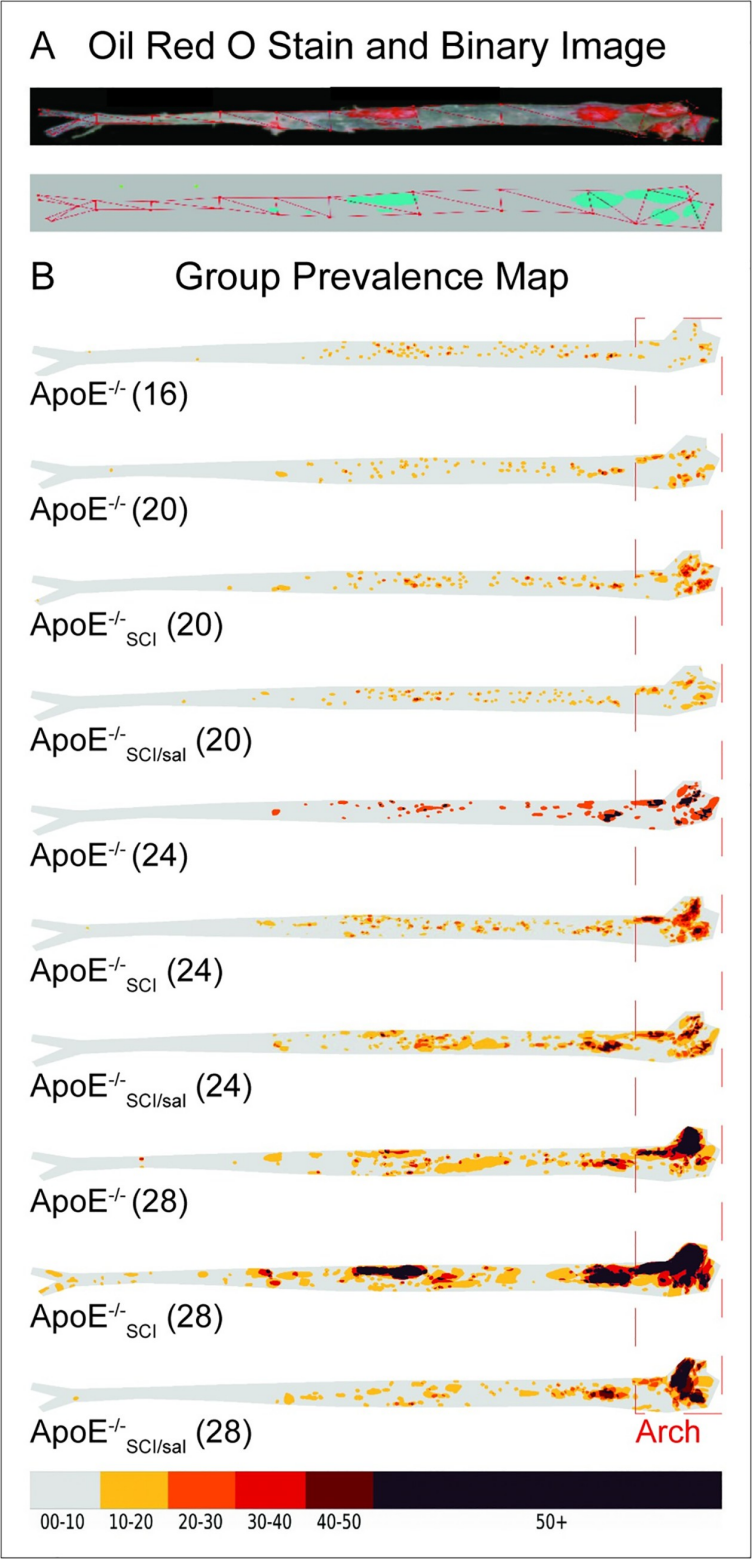
Oil Red O stained aorta and morphometric analysis of atherogenic lesion plaque lesion formation. **A.** Oil Red O (ORO) stained aorta (top) and corresponding binary image (bottom) generated from staining threshold. **B.** All groups exhibit lesions which are increasingly evident across time. Red outline indicates aortic arch region where lesion area was examined and analyzed. Numbers in brackets reflect age in weeks.

**Fig 2:**
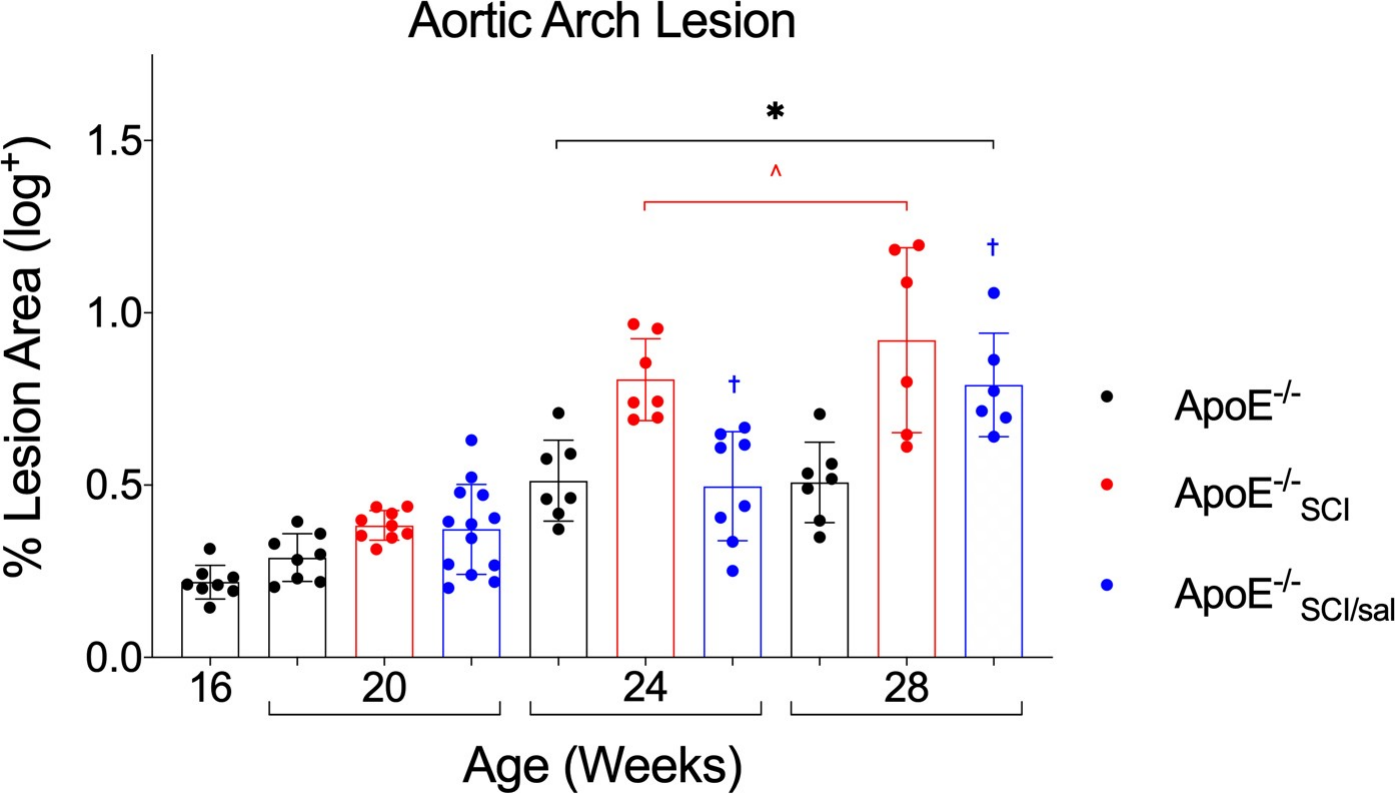
Analysis and comparison of atherosclerosis across time in *ApoE*^*-/-*^, with SCI, and Salsalate. Aortic arch lesion area in *ApoE*^*-/-*^ progressively increases over time and is significant at 24- and 28-weeks compared to 16- and 20-week (*). At 24- and 28-weeks, SCI (*ApoE*^*-/-*^_*SCI*_) lesion area is significantly greater than time-controlled *ApoE*^*-/-*^ (^). Salsalate treatment (*ApoE*^*-/-*^_*SCI/sal*_) lesion area is significantly reduced at both 24- and 28-weeks compared to SCI (*ApoE*^*-/-*^_*SCI*_, ^†^). *^^†^p < 0.05.

Atherosclerotic lesion area in the aortic arch was visualized by ORO lipid stain and between group comparisons were made from acquired cumulative lesion prevalence maps (Fig 1A and 1B). All groups exhibited lesions which are increasingly evident at later time-points (Fig 1B). Quantification of lesion area in the aortic arch show that *ApoE*^*-/-*^ exhibited significantly greater lesion area at 24- and 28- weeks compared to 16- and 20-weeks (Fig 2*). *ApoE*^*-/-*^_*SCI*_ lesion area was significantly greater than time-controlled *ApoE*^*-/-*^ at both 24- and 28- weeks (Fig 2^). Importantly, *ApoE*^*-/-*^_*SCI/sal*_ lesion area is significantly reduced compared to *ApoE*^*-/-*^_*SCI*_ at both 24- and 28-weeks, but not different from *ApoE*^*-/-*^ at these time points (Fig 2^†^).

### Body mass and lipids: Traditional AD risk factors

**Fig 3:**
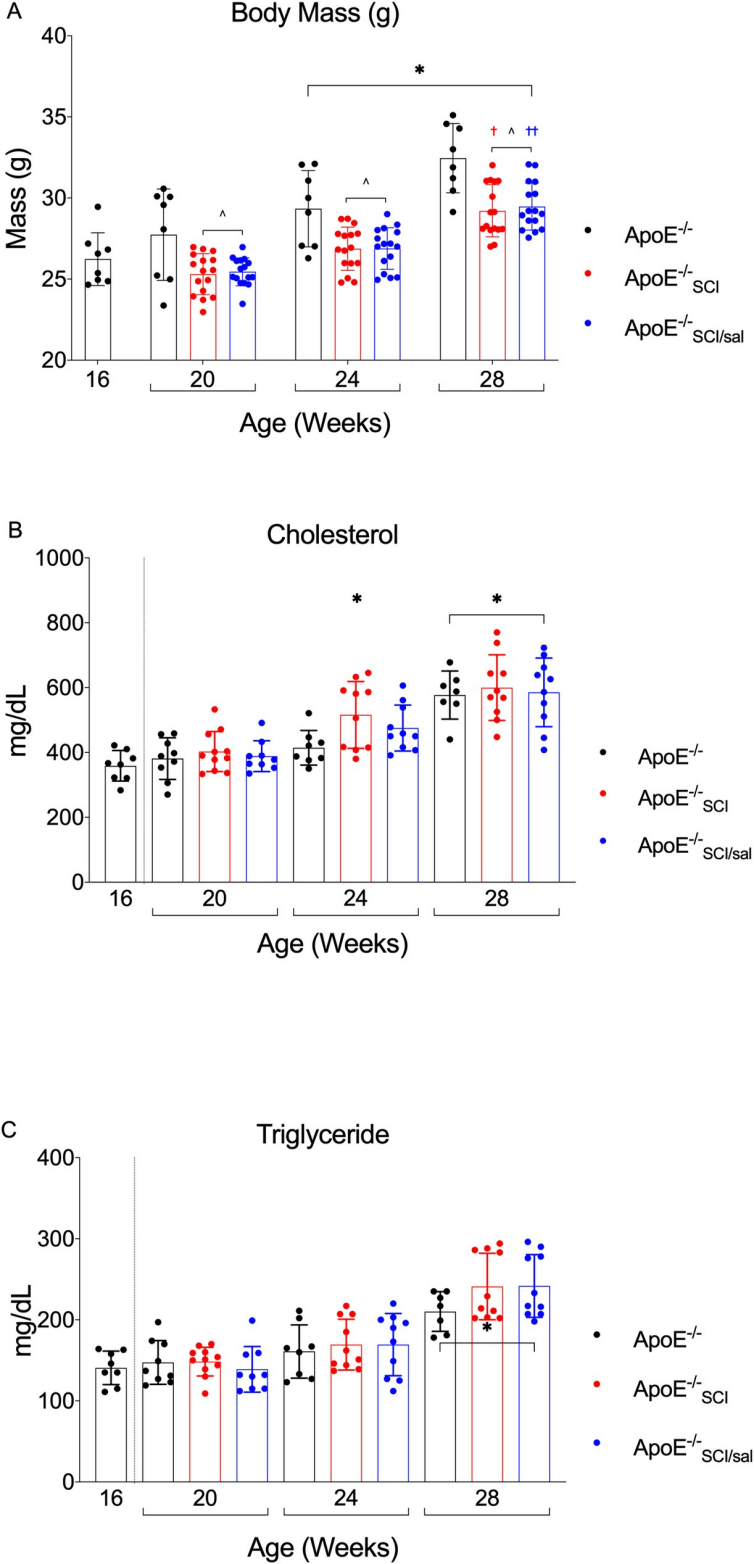
Analysis and comparison of body mass and plasma lipids across time in *ApoE*^*-/-*^, with SCI, and Salsalate. **A.** Body Mass in *ApoE*^*-/-*^ progressively increases over time and is significant at 24- and 28-weeks compared to 16- and 20-week (*). SCI (*ApoE*^*-/-*^_*SCI*_ and *ApoE*^*-/-*^_*SCI/sal*_) significantly reduces body mass at all timepoints measured compared to *ApoE*^*-/-*^ control (^). SCI groups recover body mass equally over time which is significant by 28-weeks compared to 20-weeks (^†††^). Salsalate (*ApoE*^*-/-*^_*SCI/sal*_) has no effect on body mass compared to time-controlled SCI (*ApoE*^*-/-*^_*SCI*_). **B.** Cholesterol is significantly greater in all groups at 28-weeks and with SCI (*ApoE*^*-/-*^_*SCI*_) at 24-weeks compared to all groups at 16- and 20-weeks (*). At 24-weeks, cholesterol with SCI (*ApoE*^*-/-*^_*SCI*_) is significantly greater than *ApoE*^*-/-*^ control and Salsalate treatment (*ApoE*^*-/-*^_*SCI/sal*_), and no other time-controlled differences are observed. **C.** Triglyceride is significantly greater in all groups at 28-weeks compared to all groups at previous timepoints (*). There are no differences observed between time-controlled groups. *^^†††^p < 0.05.

Body mass is significantly greater for all groups at 24- and 28-weeks compared to earlier timepoints regardless of experimental condition (Fig 3A*). *ApoE*^*-/-*^_*SCI*_ and *ApoE*^*-/-*^_*SCI/sal*_ (injury groups) exhibit significantly reduced body mass at each timepoint compared to time-controlled uninjured *ApoE*^*-/-*^ (Fig 3A^) but are not significantly different from each other at any timepoint. All groups gained weight at the same rate after 20 weeks, with both *injury* groups being significantly greater at 28-weeks compared to previous timepoints (Fig 3A^†††^).

Both plasma cholesterol and triglycerides were significantly greater in all experimental groups at 28-weeks compared to *ApoE*^*-/-*^ at previous timepoints (Figs 1C* and 3B*). Notably, at 24-weeks cholesterol in *ApoE*^*-/-*^_*SCI*_ is significantly greater than *ApoE*^*-/-*^ at 16- and 20-weeks (*), whereas neither time-controlled *ApoE*^*-/-*^ or *ApoE*^*-/-*^_*SCI/sal*_ are increased compared to any group at previous timepoints. However, at 28-weeks there is no difference in cholesterol levels between *ApoE*^*-/-*^, *ApoE*^*-/-*^_*SCI*_ or *ApoE*^*-/-*^_*SCI/sal*_. A summary of group differences for body mass, total cholesterol and triglyceride are summarized in Table 1.

**Table 1:** Mean values of body mass and serum lipids across time.

Body Mass (g) ^+/-^SEM			
_Time_^Group^	ApoE^-/-^	ApoE^-/-^_SCI_	ApoE^-/-^_SCI/sal_
Age (Weeks):			
16	26.23 ^+/-^0.57		
20	27.74 ^+/-^1.00	25.31 ^+/-^0.32	25.45 ^+/-^0.21
24	29.34 ^+/-^0.83	26.87 ^+/-^0.33	26.89 ^+/-^0.32
28	32.46 ^+/-^0.75	29.21 ^+/-^0.40	29.48 ^+/-^0.36
Cholesterol (mg/dL) ^+/-^SEM			
_Time_^Group^	ApoE^-/-^	ApoE^-/-^_SCI_	ApoE^-/-^_SCI/sal_
Age (Weeks):			
16	359.0 ^+/-^16.66		
20	381.3 ^+/-^21.42	402.9 ^+/-^18.62	388.7 ^+/-^15.76
24	414.5 ^+/-^18.91	516.2 ^+/-^32.55	475.7 ^+/-^22.44
28	577.1 ^+/-^28.10	599.8 ^+/-^32.06	585.3 ^+/-^33.42
Triglyceride (mg/dL) ^+/-^SEM			
_Time_^Group^	ApoE^-/-^	ApoE^-/-^_SCI_	ApoE^-/-^_SCI/sal_
Age (Weeks):			
16	140.9 ^+/-^ 07.29		
20	147.4 ^+/-^09.03	148.5 ^+/-^05.34	139.0 ^+/-^09.42
24	161.0 ^+/-^11.66	169.5 ^+/-^09.94	169.5 ^+/-^12.13
28	210.3 ^+/-^09.22	241.2 ^+/-^12.94	241.8 ^+/-^12.22

Values are mean ± standard error of the mean. 16-weeks represent baseline values and 28-weeks represents study endpoint.

### Inflammatory cytokines: Novel and emerging AD risk factors

Of the 23 plasma cytokines and chemokines measured 21 provided consistent results within the detectable ranges across all groups (S1A Fig). Analyses showed significant *main effect* or *group x time* interaction for 5 analytes: IL-1β, IL-6, TNFα, MCP-1, and CCL-5. Total concentration of these selected analytes, and concentration across time illustrate their mean and distribution between 1–1000 pg/mL (S1B Fig).

**Fig 4:**
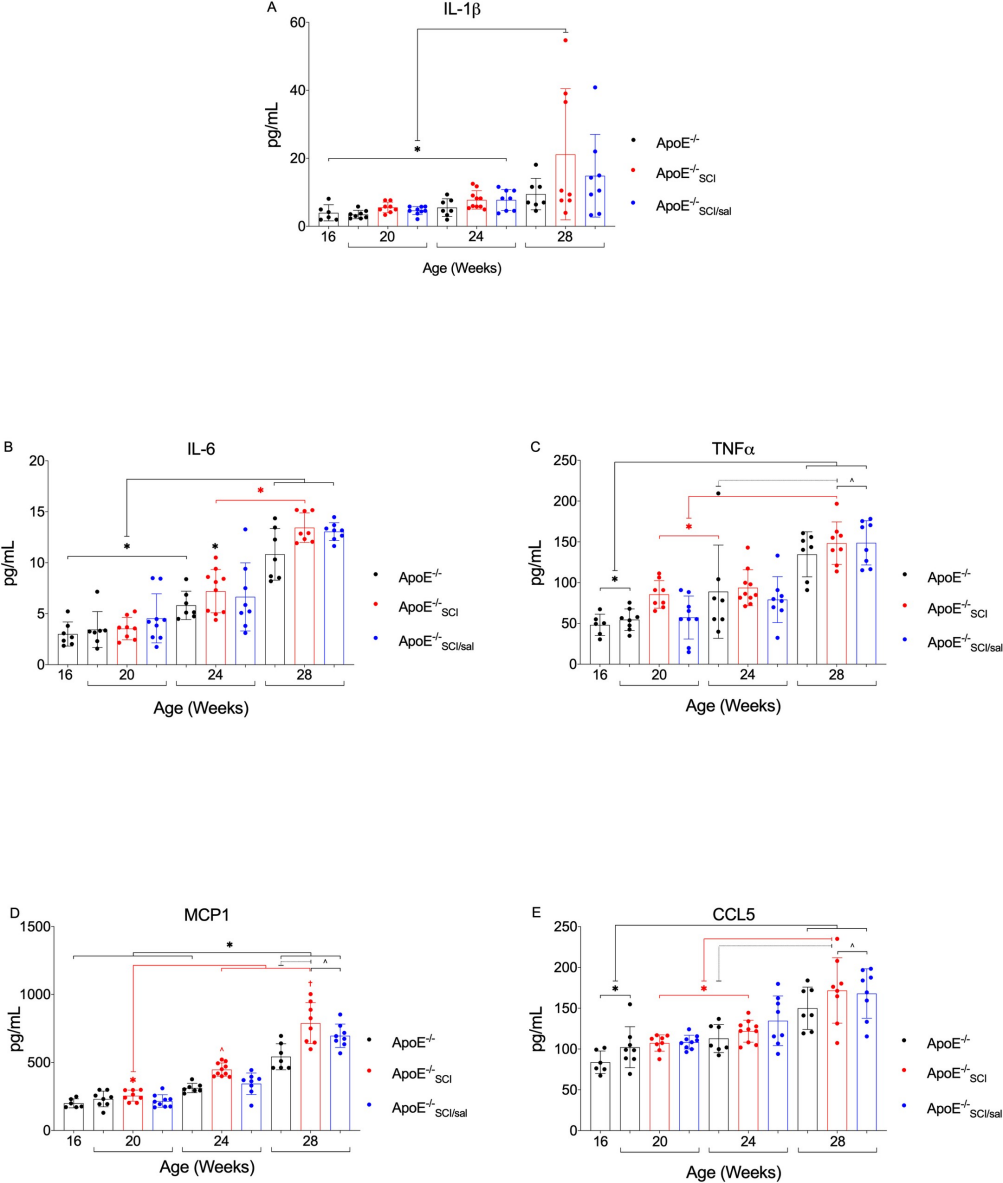
Analysis and comparison of plasma PAIC’s across time in *ApoE*^*-/-*^, with SCI, and Salsalate. **A.** IL-1β in *ApoE*^*-/-*^_*SCI*_ is significantly greater than time-controlled groups at 28-weeks and compared to all groups at previous timepoints (*). No other between group difference are observed. **B.** IL-6 is significantly greater in all groups at 28-weeks and in *ApoE*^*-/-*^_*SCI*_ at 24-weeks compared to all groups at 16- and 20-weeks (*). At 24-weeks, IL-6 in *ApoE*^*-/-*^_*SCI*_ is significantly greater than time-controlled groups, and no other time-controlled differences are observed. At 24- and 28-weeks, IL-6 in *ApoE*^*-/-*^_*SCI*_ is significantly greater compared to *ApoE*^*-/-*^_*SCI*_ at 20-weeks (*). **C.** TNFα is significantly increased in all groups at 28-weeks compared to *ApoE*^*-/-*^ at 16- and 20-weeks (*). At 28-weeks, TNFα in *ApoE*^*-/-*^_*SCI*_ but not *ApoE*^*-/-*^ is significantly greater than in *ApoE*^*-/-*^ at 24-weeks and *ApoE*^*-/-*^_*SCI*_ at 20-weeks (*). **D.** MCP-1 is significantly greater in all groups at 28-weeks compared to *ApoE*^*-/-*^ at all previous timepoints (*). At 24- and 28-weeks, MCP-1 in *ApoE*^*-/-*^_*SCI*_ is significantly greater than in *ApoE*^*-/-*^_*SCI*_ at 20-weeks (*) as well as time-controlled *ApoE*^*-/-*^ and Salsalate treatment (*ApoE*^*-/-*^_*SCI/sal*_, ^^†^). **E.** CCL-5 is significantly greater in all groups at 28-weeks compared to *ApoE*^*-/-*^ at 16- and 20-weeks (*). At 28-weeks, CCL-5 in *ApoE*^*-/-*^_*SCI*_ is significantly greater than in *ApoE*^*-/-*^_*SCI*_ at previous timepoints (*) and both inury groups (*ApoE*^*-/-*^_*SCI*_ and *ApoE*^*-/-*^_*SCI/sal*_) are significantly greater than *ApoE*^*-/-*^ (^). *^*^^†^p < 0.05.

IL-1β, IL-6 and TNF-α were similar between experimental groups at each time point and all showed a tendency to higher levels over time. MCP-1 is significantly greater in all experimental groups at 28-weeks compared to *ApoE*^*-/-*^ at all previous timepoints (Fig 4D*). At both 24- and 28-weeks, MCP-1 in *ApoE*^*-/-*^_*SCI*_ is significantly greater than time-controlled *ApoE*^*-/-*^ and *ApoE*^*-/-*^_*SCI*_ at 20-weeks (Fig 2D*^^†^). Both injury groups (*ApoE*^*-/-*^_*SCI*_ and *ApoE*^*-/-*^_*SCI/sal*_) exhibit significantly greater MCP-1 at 28-weeks compared to *ApoE*^*-/-*^ at 24-weeks (Fig 4D^).

CCL-5 is significantly greater in all experimental groups at 28-weeks compared to *ApoE*^*-/-*^ at 16- and 20-weeks (Fig 4E*). At 28-weeks, CCL-5 in *ApoE*^*-/-*^_*SCI*_ is significantly greater that in *ApoE*^*-/-*^_*SCI*_ at 20- and 24-weeks (Fig 4E*). Both injury groups (*ApoE*^*-/-*^_*SCI*_ and *ApoE*^*-/-*^_*SCI/sal*_) exhibit significantly greater CCL-5 at 28-weeks compared to *ApoE*^*-/-*^ at 24-weeks (Fig 4E^). Taken together, we observe several key time and group differences for key interleukins, including IL-1β and IL-6, and evidence for a strong injury effect on several important cytokines including TNFα, MCP-1, and CCL-5. Notably, several important effects of Salsalate are observed, in particular, mitigating significant effects of SCI on IL-1β and MCP-1.

### Regression analysis: Serum analytes as predictors of aortic arch lesion

**Fig 5:**
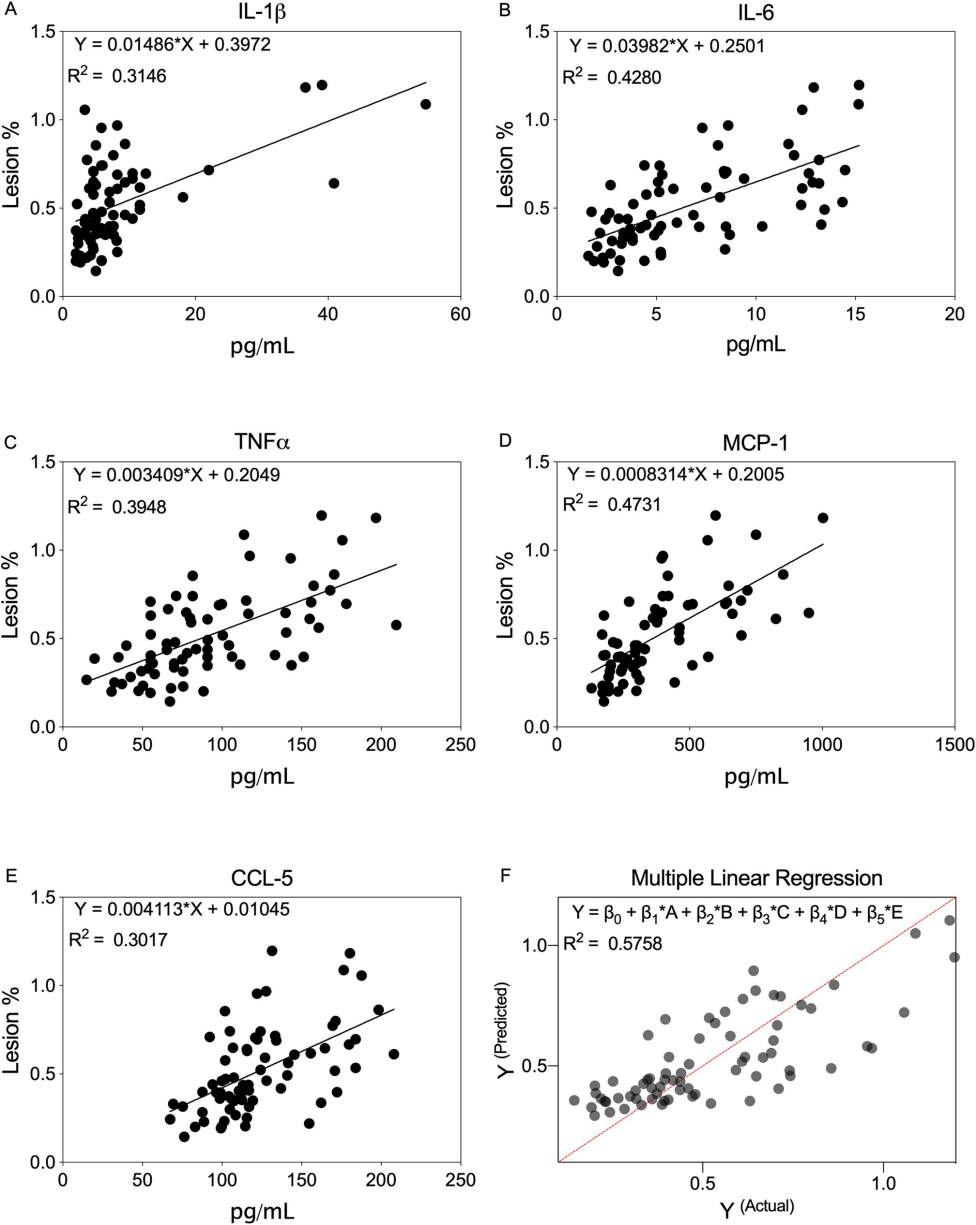
Regression analysis of PAIC levels on aortic arch lesion area in *ApoE*^*-/-*^, with SCI, and Salsalate. **A-E.** Regression equation and graphs for IL-1β, IL-6, TNFα, MCP-1, and CCL-5, respectively, indicate that each PAIC individually is a significant predictor of lesion area. **F.** Multiple regression equation and graph integrating all PAIC’s indicates that IL-1β (β_1_) and TNFα (β_3_) are significant predictors of lesion area. p < 0.05.

To determine predictors of AD, simple linear regression was performed among all experimental groups. IL-1β, IL-6, TNFα, MCP-1, and CCL-5 models determine that each is a significant and positive predictor of lesion (Fig 5A–5E, S4 Table). Corresponding coefficient estimates (β_1_) indicate that IL-6 and IL-1β are the strongest predictors of lesion. However, model *r*^*2*^ for TNFα and MCP-1 is highest amongst analytes, suggesting a better model fit of the data (S1 Table). Residual diagnostic plots demonstrate linearity of the regression models, normality, and constant variance (S2 Fig).

Given that these cytokine and chemokines may collectively mediate immune cell and inflammatory responses accompanying AD, multiple linear regression was performed incorporating all predictor variables (Fig 5F). The integrated model is a statistically significant and positive predictor of lesion, where the coefficient estimators (β_1_) for IL-1β and TNFα are significant. Of note, diagnostics for the integrated model suggest a slight positive upper-tail distribution (S2 Fig), however, this is a reasonable expectation as theoretically independent populations (i.e. experimental groups) are combined. Overall, these results illustrate that IL-1β and TNFα may best predict lesion when modelled individually or collectively.

## Discussion

This study demonstrates that SCI accelerates the rate of AD in the aortic arch in a mouse model of AD. Disease load was associated with increased levels of plasma cytokines IL-1β, IL-6 and MCP. Treatment of SCI mice with Salsalate for 4 weeks after injury resulted in a significant decrease of AD compared to non-treated SCI 8 and twelve weeks after inury. Salsalate showed modest reductions in cytokine levels (TNF, MCP, CCL5) that could account for decreased disease. It is not known if plasma cytokine levels reflect the anti-inflammatory effect of Salsalate in the vessel wall, given the drugs mechanism of action it is possible that reduced disease is related to effects on local inflammation early in the disease process during salsalate treatment that resulted in slower disease progression.

Atherosclerosis is a complex disease whose origin and progression are regulated by interaction among multiple environmental and genetic factors. The component risks of AD are consistently reported after SCI and are caused to varying degrees by 1) secondary conditions imposed by injury 2) imprudent lifestyle choices, and 3) the interplay of genetic disposition and post-injury alterations in gene expression. In particular, several reports suggest that physical deconditioning coupled with a hypercaloric diet play a major role in AD pathogenesis [1,2,52–54], as they accompany acute SCI and are known causes of obesity and insulin resistance. We have previously reported in a study of young, healthy, predominantly nonsmoking cohort of persons with intact adrenergic function (i.e., injury below the T6 spinal cord level) nearly one-third of participants satisfied authoritative guidelines for a clinical diagnosis of CMD, and nearly two-thirds qualified for risk reduction by either lifestyle intervention or drug therapy [7]. However, as described, commonly understood risks may not appropriately predict AD in SCI, and emergent disease in persons with SCI may not be identified by ‘typical’ warning signs [55]. Several human studies in SCI have used carotid intima media thickness (IMT) as a proxy for “subclinical atherosclerosis [56–58]. However, as recently reviewed [59], despite the association between carotid IMT and “hard disease” it remains unclear whether routine measurement is “useful for the detection of subclinical atherosclerosis in clinical practice”, let alone determination of extant disease. Recent attention has focused on elevated proatherogenic inflammatory biomarkers, believed to be progenitors of atherosclerosis [5]. For example, several studies indicate that plasma homocysteine [60,61] and C-reactive protein (CRP) [62–64], markers of vascular disease and atherogenesis, as well as IL-6, soluble vascular adhesion molecule (sVCAM)-1, and endothelin-1, are significantly elevated in SCI compared to neurologically intact controls [65–67]. Despite a broader view including a pro-inflammatory phenotype as a predictor of AD, we still lack an understanding of which risk factors require the greatest attention with SCI. Moreover, the extent to which AD burden is hastened by SCI is unknown.

The *ApoE*^*-/-*^ is a widely adopted model of AD, which rapidly develops atherosclerotic lesions and histopathological progression resembling human AD [39]. Notably, these mice develop fatty streaks (~10 weeks), intermediate lesions (~16 weeks) and fibrous plaques (~20 weeks), as well as altered blood lipids to an atherogenic phenotype. Disease time-course and severity can be manipulated by diet, activity, and drugs, making the *ApoE*^*-/-*^ ideal to study AD after SCI. Results here show that atherosclerotic lesions appear first in the aortic arch in young mice and progress (with aging) in the thoracic and abdominal aorta. We show *ApoE*^*-/-*^ intermediate lesions are present at 16 weeks, confirming previous literature, and that AD burden is visibly and statistically increased chronologically, although not markedly changed between 24- and 28-weeks. Importantly, our data demonstrate for the first time that SCI escalates so-called *hard* disease. The time-controlled increase in *ApoE*^*-/-*^_*SCI*_ at 24- and 28-weeks compared to *ApoE*^*-/-*^, and the fact that lesions in *ApoE*^*-/-*^_*SCI*_ at 24-weeks is greater that *ApoE*^*-/-*^ at 28-weeks, demonstrate an accelerated trajectory of AD with SCI. A point of consideration is the initial decrease in activity due to injury. Although there is an established and well-defined locomotor recovery observed in our model of injury [42,46], the initial depression in activity level due to SCI may be a contributing factor to the disease pathology. Nonetheless, this fact does not change the conclusions reached in this study. It is also important to note, that disease of the *aortic* *arch* is a significant clinical indicator of vascular events such as myocardial infarction, cerebral infarction, peripheral embolism and death, and thus was the focus of our analyses. Although there is an indication that time and injury may increase lesion in both the thoracic and abdominal aorta, these data are incomplete and unquantified. Future studies are planned to improve complete case sample size, and more comprehensively analyze lesion of the entire aortic tree. Nonetheless, the data presented in the aortic arch supports that SCI may significantly contribute to premature AD and subsequent morbidity and mortality.

The *traditional* AD risk factors, body mass, cholesterol, and triglyceride all exhibit progressive age-related increases to varying degrees in *ApoE*^*-/-*^. Acutely post-SCI (all groups), there was a rapid and expected [42] decrease in body mass which persisted at all time-points, evidence for the well reported long-term loss of metabolically active lean tissue with SCI, including muscle [42,68–74] and bone [75–81]. A gradual age-related increase in triglyceride in *ApoE*^*-/-*^ is shown, albeit significant only by 28-weeks. Effect of injury actuates an earlier significant rise in cholesterol at 24-weeks. Interpretation of these *traditional* AD risk factors are complex. Several studies suggest a strong link between ApoE and overweight/obesity [82]. *ApoE*^*-/-*^ mice accumulate less body fat content and possess smaller adipocytes compared to wild type C57BL/6 controls [83]. Moreover, the *knockout* confers resistant to body mass and adipose tissue gain even under harmful dietary conditions including high-fat, high-cholesterol, and high-sucrose [84,85]. Notably, work in humans has shown a similar correlation between ApoE and body mass index (BMI) [86]. Studies of dyslipidemia using *ApoE*^*-/-*^ mice demonstrate decreased clearance of remnant lipoproteins that leads to hypercholesterolemia and hypertriglyceridemia [87–89]. As *ApoE*^*-/-*^ alone does not produce a marked increase in body mass, our results likely reflect normal physiological and structural gain associated with advancing age, as opposed to an *obesogenic* phenotype. It is also important to note that the discrepancy between obesity in the SCI population, and body mass reduction in our model is a limitation in evaluating it as an AD risk factor. It is not surprising, however, that we observed a shift in plasma lipid profile, albeit moderate, that is proatherogenic. Most compelling is the fact that *ApoE*^*-/-*^ combined with SCI worsens hypercholesterolemia, in particular, which may be one antecedent to the increased AD burden we observe.

Inflammation is a major factor at all stages of AD. Multiple classes of cytokines play a key role in inflammation. PAIC-induced upregulation and activation of adhesion molecules and chemo-attractants, and migration and infiltration of immune cells, collectively contribute to AD progression [90,91]. As such, the robust cumulative effect of PAIC’s on AD is well recognized, as is the difficulty in discerning the effect of each one individually. Therefore, exploiting a high-throughput automated method allowed us to analyze and determine global patterns of PAIC change in this study. Our data provide evidence that physiological levels of several key cytokines are upregulated with time in *ApoE*^*-/-*^ and that injury worsens their expression profile. For example, elevated IL-1β with SCI shown here, advance previous studies with *ApoE*^*-/-*^ in which IL-1β is shown to worsen AD via upregulation of adhesion molecules and macrophage activation in the vascular wall [92–95]. Moreover, activation of IL-1β in AD is linked to the NLRP3 inflammasome [96], and we have reported NLRP3 inflammasome formation and IL-1β activation in visceral adipose tissue and pancreas [44], known intermediaries of metabolic diseases. A previous clinical trial has shown that targeting IL-1β reduced cardiovascular events [97], and one recent cross-sectional study reported that IL-1β levels were reduced in an SCI cohort with an anti-inflammatory intervention [98], These studies suggest that treatments targeted at IL-1β may have efficacy in AD and SCI. Similarly, our results indicate a greater overall, and earlier chronological rise in IL-6 with SCI. IL-6’s role in AD may be pathological [99] or protective [100] depending on the stage of atherogenesis. [Reviewed in [101,102]] In a previous study in humans with SCI examining subclinical atherosclerosis–as evaluated by carotid intima-media thickness (IMT)–no relationship with serum IL-6 was found [56], although the authors report an increase in leukocyte-derived IL-6. Nonetheless, our expression analysis and prediction models indicate a concurrent increase with aortic arch lesion and a positive relationship between the two, suggesting a proatherogenic effect.

Similarly, TNFα, MCP-1, and CCL-5 mediate pro-inflammatory effects via mechanisms associated with increased vascular injury leading to AD and myocardial infarction [103,104]. AD progression is directly correlated with TNFα production in In *ApoE*^*-/-*^ [105], and in clinical studies, circulating levels of TNFα and soluble TNFRs are independent predictors of mortality in patients with heart failure [106]. However, in several seminal trials of anti-TNFα therapy, unexpected and worsened outcomes resulted [107,108]. It is now better appreciated that global/systemic TNFα inhibition may dysregulate divergent adverse and protective inflammatory responses conferred by specific TNFα receptors [104]. Interestingly, TNFα also upregulates the potent chemo-attractants MCP-1 and CCL-5 [109–112]. MCP-1 was the first chemokine implicated in AD pathogenesis and identified in both mouse and human atherosclerotic lesions [113,114]. CCL-5 is also expressed by a variety of immune and vascular cells in both mouse and human atherosclerotic lesions [115,116]. Not surprisingly, in *ApoE*^*-/-*^ we show a progressive increase in TNFα, MCP-1, and CCL-5 which are significant by 28-weeks. Collectively, not only do we observe a strong effluence of several potent PAIC’s in a progressive time-course in *ApoE*^*-/-*^, we have demonstrated that our model of SCI can incite and accelerate increases in their serum expression. Aligned with our initial hypothesis, each of these PAIC’s were able to predict aortic lesion individually, and when incorporated into an ‘*inflammatory*’ model, IL-1β and TNFα may be forerunners of AD predictions. In this way, their incorporation into more robust models, including lipid and other proatherogenic predictors may better forecast AD risk.

Activation of the transcription factor NF-κB is known to play a key role in the proatherogenic effects of PAIC’s. NF-κB as a primary mediator of vascular disorders has extensively been reviewed [117], linked to diverse processes and a coordinated inflammatory response. Given the interplay of these pathogenic factors and that CMD *risk clustering* is evident in SCI, targeting NF-κB signaling is an attractive treatment strategy to mitigate these metabolic and disease risks. Low-dose salicylates (eg. Aspirin 81 mg/day) are shown to have anti-thrombotic effects, and higher doses (≥ 2 mg/day) are shown to have systemic anti-inflammatory effects [38,117]. Randomized clinical trials have shown that Salsalate improves glycemic and inflammatory markers in subjects with diabetes or related risk factors (i.e. obesity) [32,33,118,119]. The efficacy of Salsalate is less conclusive in studies evaluating vascular dysfunction [120] and extant coronary artery disease [121], however, the authors themselves discuss important limitations including trial duration, study power, administration with advanced disease and in concert with other guideline-directed pharmacotherapeutics. Notably, an important component of AD is platelet-mediated, where early inflammatory events may stimulate platelet attachment [122,123]. However, Salsalate has no effect on platelet activity [32,124–126], supporting that appropriate adjunctive treatment may be critical.

In general, Salsalate has systemic anti-inflammatory effects [Reviewed in [126]], and in particular, has been shown to improve atherosclerotic inflammatory responses [36], and pro-atherogenic lipid profiles [37]. Animal and *in vitro* studies using Salsalate have also shown favorable results with earlier intervention with respect to vascular dysfunction and inflammatory response [34,36]. Given these known effects of Salsalate on atherosclerosis, the focus of this study was directed at i. defining atherosclerosis *in SCI*–which has not been previously shown and ii. examining the effect of salsalate on the impact of SCI, as a means of streamlining *between group* comparisons and study resources. However, we note the lack of an *ApoE*^*-/-*^ + Salsalate comparison group as a limitation in comprehensively evaluating differences between experimental groups. In this report, we did not find that Salsalate–i.e. *ApoE*^*-/-*^_*SCI/sal*_−significantly reduced PAIC’s examined when compared to SCI without treatment–i.e. *ApoE*^*-/-*^_*SCI*_ alone. By 28-weeks, an argument could be made that all inflammatory analytes are observably lower, although it would be remiss to attribute this as a treatment effect. More likely is that the severity of SCI superimposed on a *pro-inflammatory* genotype resulted in a chronic–and robust–inflammatory response that could not be significantly thwarted. Importantly, the treatment schedule here lasted for only the first 30 days post-SCI which may be insufficient to i. overcome the early effects of SCI and ii. mitigate chronic time-related inflammation. Nonetheless, when examining the aortic arch, we do in fact demonstrate that Salsalate significantly reduces lesions at 24- and 28-weeks when compared to untreated SCI. This may represent a collective acute-phase effect on inflammatory machinery that slows–or delays–the processes involved in lesion formation. Given the current treatment schedule, it could be predicted that Salsalate only slows the progression of atherosclerosis and no differences would be notable if the observation window were extended beyond 28-weeks, when lesion load typically becomes saturated in the aortic arch. Longer studies with dose-scaling and/or longer treatment may address this, although it should be noted that a previously reported clinical study raised concern over deleterious endothelial function with long-term, high-does Salsalate therapy [38]. Taken together, our results are noteworthy insofar as they provide direct evidence that Salsalate attenuates aortic lesion and suggest a basis for future studies that more closely examines treatment dose, timing, and the effect on inflammatory analytes.

## Conclusion

In this study we provide evidence that SCI accelerates systemic dyslipidemia, inflammation and atherosclerotic plaque. We show the potential for Salsalate to mitigate pro-inflammatory and proatherogenic consequences of SCI, and introduce PAIC’s that may effectively predict AD. Continued studies will clarify the extent to which Salsalate is a feasible treatment strategy for secondary health complications that accompany SCI, and better evaluate whether PAIC biomarkers are suitable to integrate into clinical models of AD risk.

## Supporting information

S1 Fig Multiplex screening profile of cytokine and chemokine immune biomarkers in *ApoE*^*-/-*^, with SCI, and Salsalate. **A.** Serum levels (illustrated as log values) of 21 analytes (shown) indicate global expression patterns for each, detectable across all timepoints and experimental groups. **B.** Select analytes with significant time and/or between group differences: IL-1β, IL-6, TNFα, MCP-1, and CCL-5 each exhibit detectable concentrations between 1–1000 pg/mL (left graph) and an increase in concentration across time (right graph).
(TIFF)

S2 Fig Regression analysis diagnostic plots. Residuals for IL-1β, IL-6, TNFα, MCP-1, and CCL-5, respectively, illustrating general assumptions of linear regression: *Residual vs Fitted*–linearity of the model; *Normal QQ*–normal distribution of residuals; *Scale-Location*–constant variance; and *Residuals vs Leverage*–extreme values (see S4 Table for model coefficients and statistical tests).
(TIF)

S3 Fig Normal distribution plots for dependent variables. Quantile-quantile (QQ) probability scatterplots for: body mass, aortic arch lesion, cholesterol, triglyceride, IL-1β, IL-6, TNFα, MCP-1, and CCL-5 –illustrating linearity of the sample distribution (see S2 Table for statistical tests).
(TIFF)

S1 Table Regression analysis coefficient estimates and statistical tests: IL-1β, IL-6, TNFɑ, MCP-1, CCL-5, and integrated model. (XLSX)

S2 Table Descriptive summary statistics for dependent variables: Body mass, cholesterol, triglyceride, lesion area, IL-1β, IL-6, TNF⍺, MCP-1, and CCL-5. (XLSX)

S3 Table Shapiro Wilkes (W) normality test for dependent variables: Body mass, cholesterol, triglyceride, lesion area, IL-1β, IL-6, TNF⍺, MCP-1, and CCL-5. (XLSX)

S4 Table Analysis of variance (ANOVA) table for dependent variables: Body mass, cholesterol, triglyceride, lesion area, IL-1β, IL-6, TNF⍺, MCP-1, and CCL-5. (XLSX)
