# Supplementary figures and images for: Cardiometabolic risks and atherosclerotic disease in ApoE knockout mice: Effect of spinal cord injury and Salsalate anti-inflammatory pharmacotherapy
Source: PLoS One. 2021 Feb 24;16(2):e0246601. doi: 10.1371/journal.pone.0246601 (PMC7904230; doi:10.1371/journal.pone.0246601)

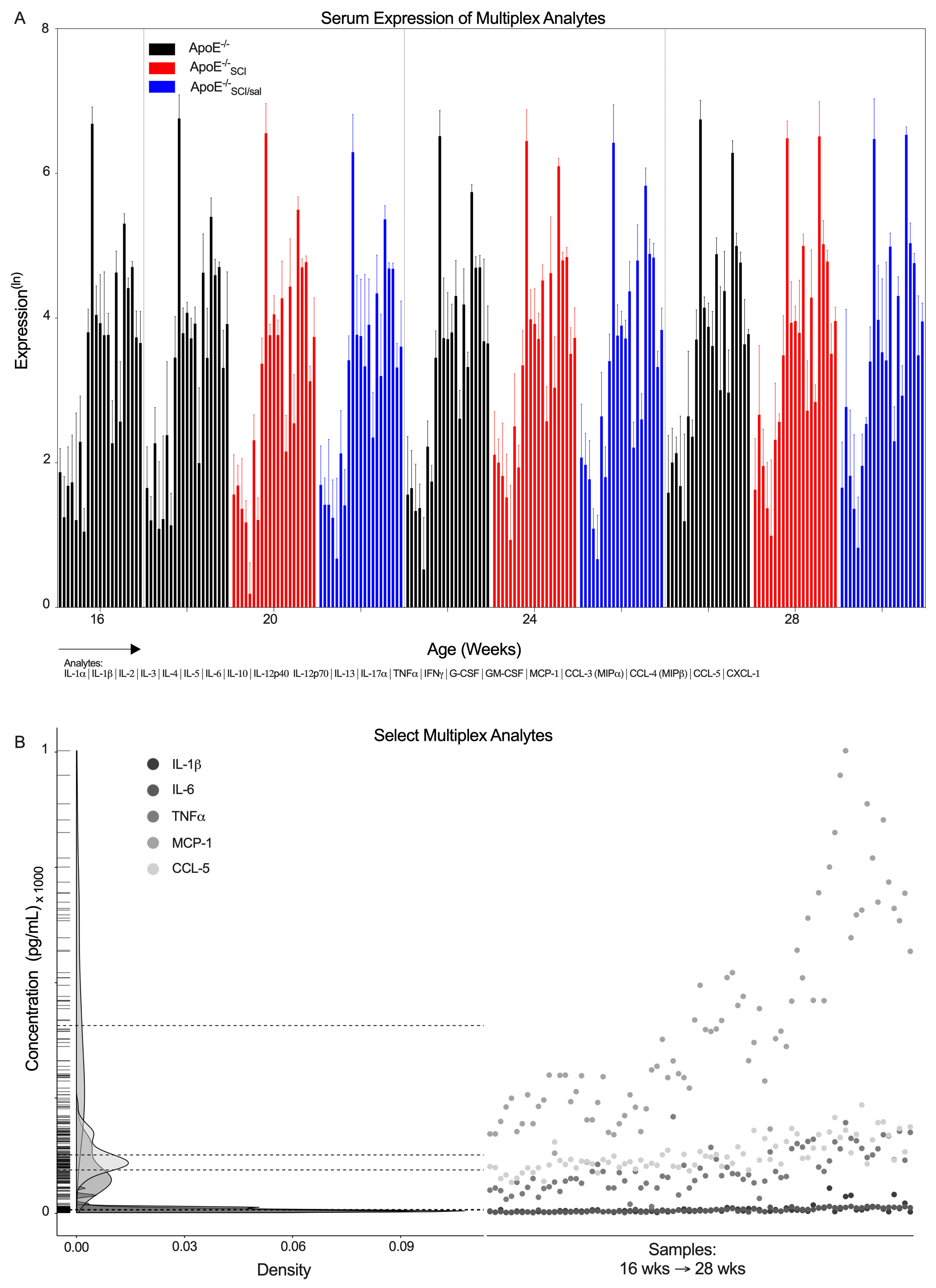

Supplement: S1 Fig — A. Serum levels (illustrated as log values) of 21 analytes (shown) indicate global expression patterns for each, detectable across all timepoints and experimental groups. B. Select analytes with significant time and/or between group differences: IL-1β, IL-6, TNFα, MCP-1, and CCL-5 each exhibit detectable concentrations between 1–1000 pg/mL (left graph) and an increase in concentration across time (right graph). (TIFF) [file pone.0246601.s001.tiff]

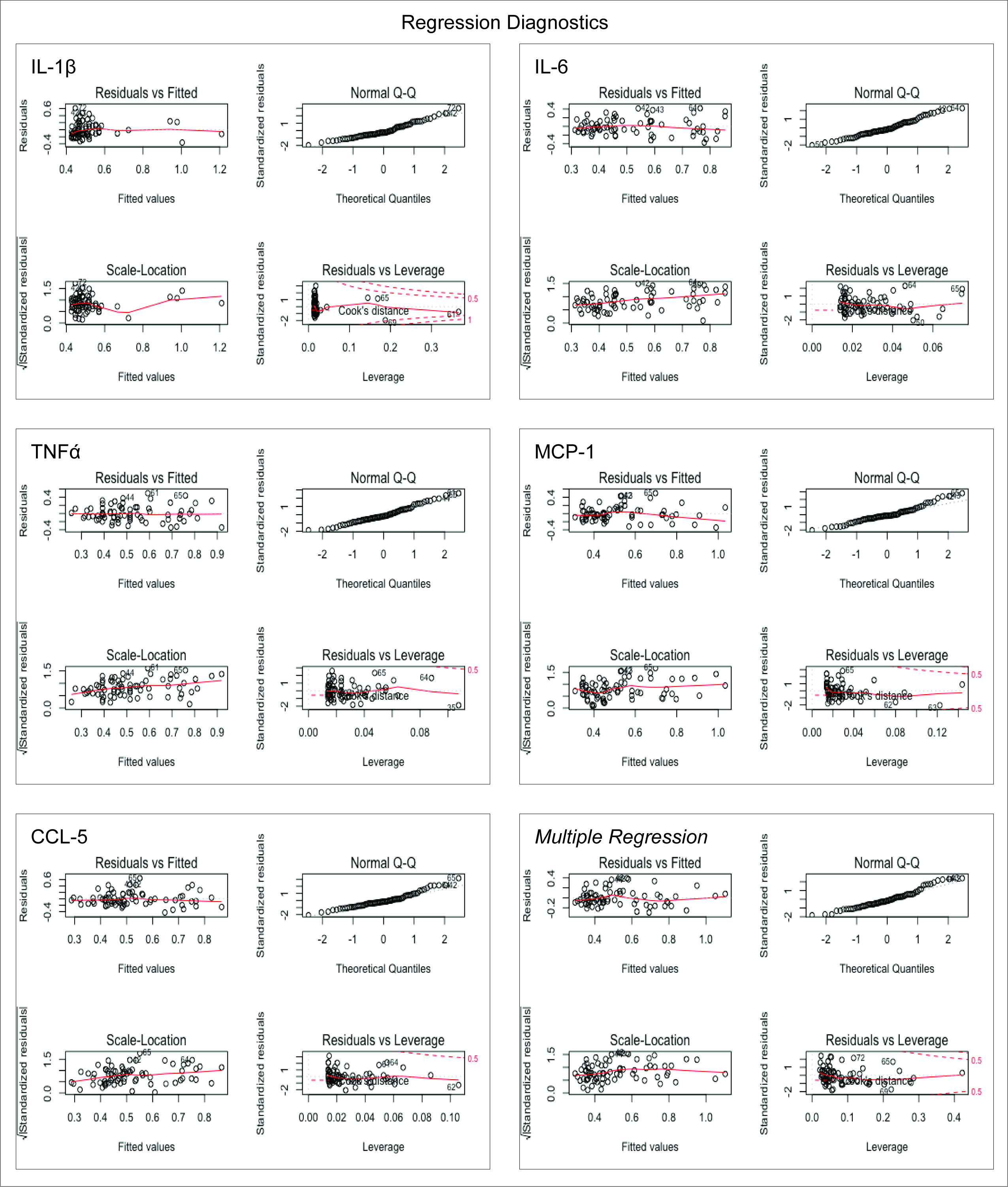

Supplement: S2 Fig — Residuals for IL-1β, IL-6, TNFα, MCP-1, and CCL-5, respectively, illustrating general assumptions of linear regression: Residual vs Fitted–linearity of the model; Normal QQ–normal distribution of residuals; Scale-Location–constant variance; and Residuals vs Leverage–extreme values (see S4 Table for model coefficients and statistical tests). (TIF) [file pone.0246601.s002.tif]

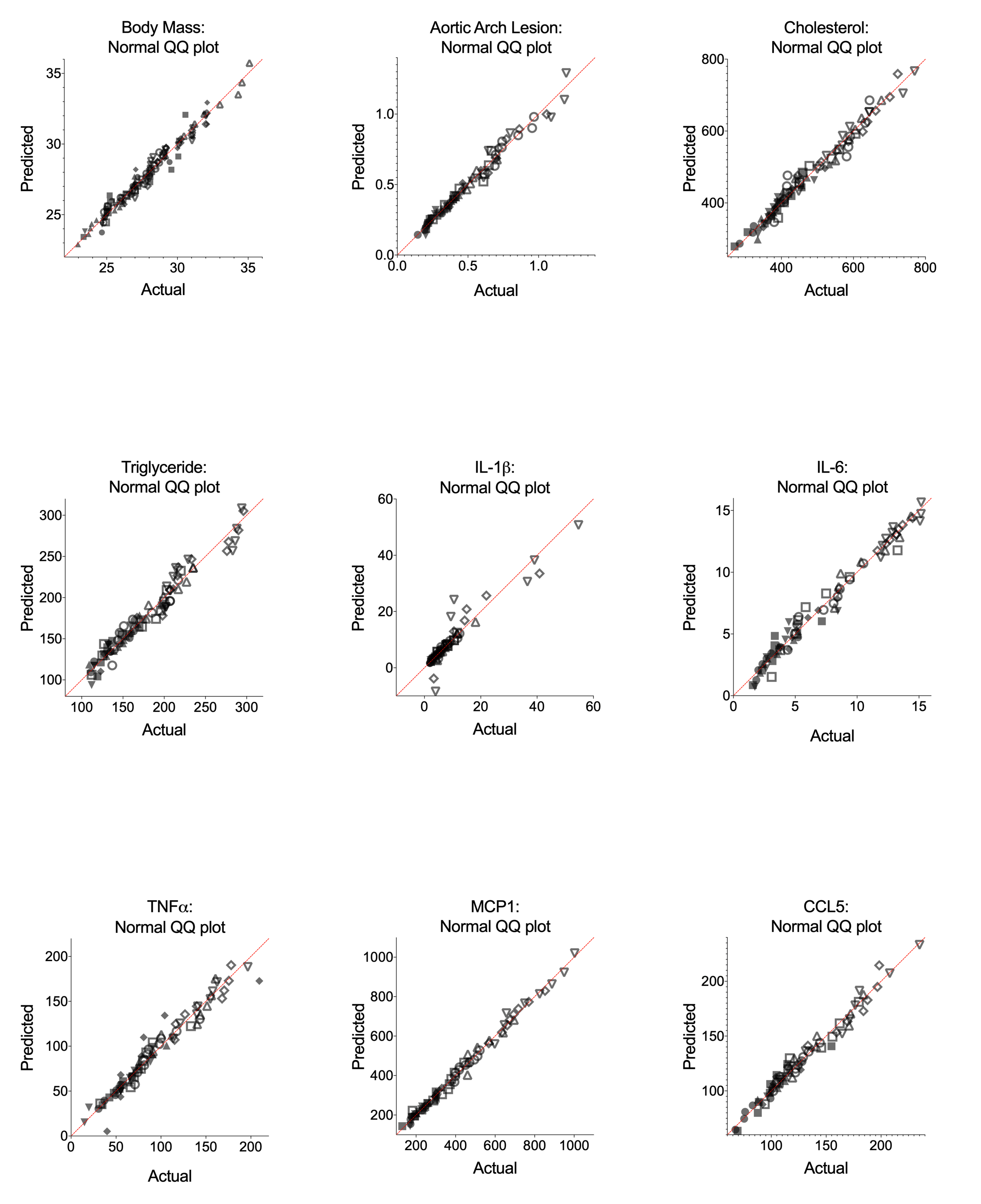

Supplement: S3 Fig — Quantile-quantile (QQ) probability scatterplots for: body mass, aortic arch lesion, cholesterol, triglyceride, IL-1β, IL-6, TNFα, MCP-1, and CCL-5 –illustrating linearity of the sample distribution (see S2 Table for statistical tests). (TIFF) [file pone.0246601.s003.tiff]
